# Combined Use of Serum Adiponectin and Tumor Necrosis Factor-Alpha Receptor 2 Levels Was Comparable to 2-Hour Post-Load Glucose in Diabetes Prediction

**DOI:** 10.1371/journal.pone.0036868

**Published:** 2012-05-16

**Authors:** Yu-Cho Woo, Annette W. K. Tso, Aimin Xu, Lawrence S. C. Law, Carol H. Y. Fong, Tai-Hing Lam, Su-Vui Lo, Nelson M. S. Wat, Bernard M. Y. Cheung, Karen S. L. Lam

**Affiliations:** 1 Department of Medicine, The University of Hong Kong, Pokfulam, Hong Kong; 2 Research Centre of Heart, Brain, Hormone and Healthy Aging, The University of Hong Kong, Pokfulam, Hong Kong; 3 Department of Community Medicine, The University of Hong Kong, Pokfulam, Hong Kong; 4 Hospital Authority, Kowloon, Hong Kong; The University of Hong Kong, Hong Kong

## Abstract

**Background:**

Adipose tissue inflammation and dysregulated adipokine secretion are implicated in obesity-related insulin resistance and type 2 diabetes. We evaluated the use of serum adiponectin, an anti-inflammatory adipokine, and several proinflammatory adipokines, as biomarkers of diabetes risk and whether they add to traditional risk factors in diabetes prediction.

**Methods:**

We studied 1300 non-diabetic subjects from the prospective Hong Kong Cardiovascular Risk Factor Prevalence Study (CRISPS). Serum adiponectin, tumor necrosis factor-alpha receptor 2 (TNF-α R2), interleukin-6 (IL-6), adipocyte–fatty acid binding protein (A-FABP) and high-sensitivity C-reactive protein (hsCRP) were measured in baseline samples.

**Results:**

Seventy-six participants developed diabetes over 5.3 years (median). All five biomarkers significantly improved the log-likelihood of diabetes in a clinical diabetes prediction (CDP) model including age, sex, family history of diabetes, smoking, physical activity, hypertension, waist circumference, fasting glucose and dyslipidaemia. In ROC curve analysis, “adiponectin + TNF-α R2” improved the area under ROC curve (AUC) of the CDP model from 0.802 to 0.830 (P = 0.03), rendering its performance comparable to the “CDP + 2-hour post-OGTT glucose” model (AUC  = 0.852, P = 0.30). A biomarker risk score, derived from the number of biomarkers predictive of diabetes (low adiponectin, high TNF-α R2), had similar performance when added to the CDP model (AUC  = 0.829 [95% CI: 0.808–0.849]).

**Conclusions:**

The combined use of serum adiponectin and TNF-α R2 as biomarkers provided added value over traditional risk factors for diabetes prediction in Chinese and could be considered as an alternative to the OGTT.

## Introduction

Diabetes mellitus is an increasing global health problem. In a recent study [1], the prevalence of diabetes in the Chinese population in 2008 had increased to 9.7% among adults aged 20 years or older. Another 12.9% of the subjects in this study had impaired glucose tolerance (IGT), known to be associated with a highly elevated risk of diabetes development among various populations including Hong Kong Chinese [2]. Without appropriate public health measures, it is anticipated that the increase in diabetes prevalence in China will reach epidemic dimensions in the near future. On a brighter note, it has been shown in long-term prospective studies that lifestyle interventions can delay or even prevent the onset of type 2 diabetes in high risk individuals including subjects with IGT [3], [4], [5]. To identify the presence of IGT, however, it is necessary to perform an oral glucose tolerance test (OGTT) which is cumbersome and not consistently reproducible. From a public health point of view, it would be very helpful if alternative, more easily assessed biomarkers with high predictive value for diabetes development can be identified.

Adipose tissue inflammation and dysregulated adipokine secretion have been implicated in obesity-related insulin resistance and type 2 diabetes [6], [7]. High levels of the pro-inflammatory biomarkers, such as interleukin-6 (IL-6), tumor necrosis factor-alpha (TNF-α) or its soluble receptor tumor necrosis factor-alpha receptor 2 (TNF-α R2), and C-reactive protein (CRP), are found in obese individuals. In Chinese, high level of high sensitive C-reactive protein (hsCRP) has been shown to independently predict the deterioration of glycaemia [8]. In addition, IL-6 and TNF-α, the upstream cytokines of C-reactive protein in the inflammatory cascades, have also been linked with an increased risk of type 2 diabetes in a previous report [9], although other studies have shown inconsistent results [10], [11], [12]. Our group has previously demonstrated that a high baseline level of adipocyte fatty acid binding protein (A-FABP), a pro-inflammatory adipokine, is also predictive of type 2 diabetes in a Chinese cohort [13]. Among the various biomarkers, adiponectin, an anti-inflammatory, insulin-sensitizing adipokine with reduced expression in obesity, has been consistently linked with protection from type 2 diabetes [10], [11], [12], [14]. We have demonstrated, in a previous nested case-control study, that hypoadiponectinaemia could predict persistent hyperglycaemia in Chinese [14]. In this 5-year prospective study of a population-based Chinese cohort, we examined the association of the above obesity-related biomarkers with diabetes development in the Chinese population and investigated if any of these biomarkers have the potential to replace the OGTT for risk prediction.

## Methods

### Ethics

The study was approved by the ethics committee of the Faculty of Medicine, University of Hong Kong. All subjects gave written informed consent.

### Participants

The Hong Kong Cardiovascular Risk Factor Prevalence Study (CRISPS) is a population-based, long-term follow-up study on the prevalence of cardiovascular risk factors in Hong Kong. [2], [14], [15], [16] In 1995–1996 (CRISPS1), 2,895 unrelated Chinese subjects were selected randomly by their telephone numbers to undergo a comprehensive assessment, including a 75 g OGTT in all subjects not on antidiabetic medications. Subjects were contacted for reassessment in 2000–2004 (CRISPS2) and in 2005–2008 (CRISPS3). The current study included only the non-diabetic subjects identified in CRISPS2 (baseline for this study), as defined by the World Health Organization 1998 criteria [17]. 1300 subjects who attended both CRISPS2 (baseline visit) and CRISPS3 (follow-up visit) and had complete baseline anthropometric and biochemical data were included for analysis. Subjects who had FG ≥7 mmol/L or 2-hG ≥11.1 mmol/L [17] at CRISPS3 or had been diagnosed to have diabetes between CRISPS2 and CRISPS3 were considered as incident cases of type 2 diabetes. At each attendance, medical histories were reviewed in detail. Anthropometric and biochemical parameters were measured as described previously [2], [14], [15], [16]. The presence of hypertension was defined as blood pressure ≥130/85 mmHg or receiving regular antihypertensive treatment. The presence of dyslipidaemia was defined as having high triglyceride (fasting triglycerides ≥1.69 mmol/L), low HDL-C (fasting HDL-C <1.29 mmol/L in women and <1.04 mmol/L in men), high LDL-C (fasting LDL-C ≥3.4 mmol/L) [18] or taking lipid lowering agents. Insulin resistance was estimated using the homeostasis model assessment index of insulin resistance (HOMA-IR), calculated by the formula (FG in mmol/L × fasting insulin in mIU/L /22.5) [19].

### Biochemical Assessments

Serum levels of the biomarkers were measured from stored serum samples collected at CRISPS2 (baseline of this study). Serum A-FABP [13], IL-6 and TNF-α R2 were measured with commercial ELISA kits (BioVendor – Laboratory Medicine, Modrice, Czech Republic for A-FABP; Bender MedSystems GmbH, Vienna, Austria for IL-6; R&D Systems, Inc., Minneapolis, USA for TNF-α R2). hsCRP and total adiponectin levels were measured using in-house sandwich ELISA assays established in our laboratory as described previously [14].

### Statistical Analyses

All statistical analyses were performed with SPSS Statistics 19 (SPSS, Chicago, IL). Results are presented as means ± SD or medians (interquartile range, IQR) as appropriate. Data that were not normally distributed, as determined using Kolmogorox-Smirnov test, were natural-logarithmically transformed to obtain near normality before analysis. A-FABP, IL-6, TNF-α R2 and adiponectin levels showed gender-specific dimorphisms and were sex-adjusted. Differences in baseline characteristics with subsequent glycaemia status were compared using χ2 tests for categorical variables and one-way ANOVA for continuous variables. The level of each biomarker was divided into high and low categories by an optimal cutoff derived from Youden Index (sensitivity + specificity −1) [20]. Multiple logistic regression was applied to estimate the odds ratio (OR) and 95% confidence interval (CI) of each biomarker by comparison of the two categories for incident diabetes. We used the low level as the reference for TNF-α R2, IL-6, hsCRP and A-FABP; and the high level as reference for adiponectin. In multivariate analyses, we adjusted for clinical parameters and conventional risk factors including age, sex, family history of diabetes in first degree relatives, smoking status, physical activity, hypertension, waist circumference (WC), fasting glucose (FG) and dyslipidaemia. Log-likelihood ratio test was used to compare the likelihood of incident diabetes before and after addition of one, followed by multiple, biomarker levels to the clinical diabetes prediction model (CDP), which included the aforementioned clinical parameters and diabetes risk factors. A biomarker risk score was counted by the number of biomarkers predictive of diabetes. Receiver Operating Characteristic (ROC) curves were used to assess the performance of different methods for diabetes risk prediction. The area under ROC curves (AUC) were compared with a nonparametric approach as described by DeLong et al [21]. Sensitivities, specificities, and positive and negative predictive values for the different prediction models were determined. Two-sided p-values less than 0.05 were considered statistically significant.

## Results

Seventy-six of 1300 subjects (5.85%) had developed type 2 diabetes when reassessed after a median interval of 5.3 (inter-quartile range: 4.59–5.80) years. Table 1 shows that at baseline, compared to those without incident diabetes at follow-up, subjects with incident diabetes were significantly older, more obese, had greater BMI and WC, higher FG, 2 h-glucose, insulin, HOMA-IR, triglyceride and LDL-C, but lower level of HDL-C. They were more likely to have hypertension, dyslipidaemia, IGT or impaired fasting glucose (IFG) [17]. For the serum biomarkers, subjects with incident diabetes had lower levels of adiponectin (P = 0.001), but higher levels of hsCRP (P<0.001), IL-6 (P = 0.014), TNF-α R2 (P<0.001) and A-FABP (P<0.001).

**Table 1:** Baseline clinical and biochemical characteristics of subjects with and without incident type 2 DM in 5.3 years.

Baseline parameters	DM	Non-DM	p-value
N	76	1224	–
Age (year)	56.6±10.5	49.8±10.9	<0.001
Female (%)	50.0	55.2	0.375
Current/Former smoker (%)	31.6	23.9	0.131
Physical activity (%)^a^	26.0	30.8	0.388
Family history of diabetes	21.1	16.7	0.329
Central Obesity (%)	46.1	23.8	<0.001
Waist circumference (cm)	–	–	<0.001^b^
Male	89.0±9.02	82.8±8.46	–
Female	81.5±8.91	74.9±8.67	–
Body Mass Index (kg/m^2^)	25.8±3.5	23.7±3.3	<0.001
Hypertension (%)	35.5	20.6	0.002
Systolic blood pressure (mmHg)	–	–	<0.001^c^
With hypertensive treatment	140.1±25.9	136.0±16.8	–
Without hypertensive treatment	126.7±17.4	118.5±17.0	–
Diastolic blood pressure (mmHg)	–	–	0.001^c^
With hypertensive treatment	88.1±10.1	82.3±10.3	–
Without hypertensive treatment	78.0±8.35	74.0±10.2	–
IGT/IFG (%)	71.1	24.1	<0.001
Fasting glucose (mmol/L)	5.4±0.6	5.0±0.5	<0.001
2-hour glucose (mmol/L)	8.4±1.6	6.5±1.7	<0.001
Fasting insulin (mIU/L)^d^	8.75(6.63–15.0)	6.95(5.08–9.80)	<0.001
HOMA-IR^d^	2.16(1.49–3.54)	1.53(1.11–2.24)	<0.001
Dyslipidaemia (%)	77.3	60.0	0.003
Total cholesterol (mmol/L)^e^	5.48±1.09	5.25±0.87	0.032
LDL cholesterol (mmol/L)^e^	3.45±0.97	3.25±0.77	0.036
HDL cholesterol (mmol/L)^e^	1.32±0.33	1.43±0.38	0.014
Triglycerides (mmol/L)^de^	1.3(0.9–2.0)	1.1(0.8–1.5)	<0.001
Adiponectin (ug/ml)^d^	–	–	0.001^b^
Male	4.53(3.07–5.93)	5.88(3.78–9.19)	–
Female	6.73(4.18–9.47)	8.20(5.74–11.8)	–
TNF-alpha R2 (ng/ml)^d^	–	–	<0.001^b^
Male	2.24(1.89–2.64)	1.96(1.69–2.31)	–
Female	2.06(1.56–2.53)	1.76(1.52–2.07)	–
hsCRP (mg/L)^d^	1.24(0.65–2.12)	0.64(0.30–1.39)	<0.001
Interlukin-6 (pg/ml)^d^	–	–	0.014^b^
Male	0.84(0.48–1.26)	0.58(0.36–0.86)	–
Female	0.59(0.37–0.88)	0.51(0.35–0.75)	–
A-FABP (ng/ml)^d^	–	–	<0.001^b^
Male	26.36(16.58–36.69)	18.05(13.28–24.00)	–
Female	31.16(23.8–36.93)	22.03(16.29–30.20)	–

Mean ± SD, median (interquartile-range), or percentage as appropriate.

a Physical activity: active if having moderate intensity exercise for at least 30 minutes in one month. ^b^Sex-adjusted; ^c^Adjusted for hypertensive treatment; ^d^Log transformed before analysis. ^e^Excluded subjects on lipid treatment;

Central obesity: waist circumference ≥90 cm (M)/80 cm (F).

Hypertension: systolic blood pressure ≥140 mmHg, diastolic blood pressure ≥90 mmHg, or on hypertensive treatment.

Dyslipidaemia: triglycerides ≥1.7 mmol/L, HDL cholesterol <1.0 mmol/L (M)/1.3 mmol/L (F), LDL cholesterol ≥3.4 mmol/L, or on lipid treatment.

The levels of biomarkers were divided into low and high categories by their respective optimal cutoffs, i.e. 5.94 ug/ml (M) and 6.03 ug/ml (F) for adiponectin; 2.13 ng/ml (M) and 2.05 ng/ml (F) for TNF-α R2; 21.72 ng/ml (M) and 26.33 ng/ml (F) for A-FABP; 0.71 pg/ml (M) and 0.34 pg/ml (F) for IL-6, and 0.62 mg/L for hsCRP. Each of the five serum biomarkers was tested individually by multivariate analysis to examine for its independent association with incident diabetes, with adjustments made for age, sex, family history of diabetes, smoking status, physical activity, hypertension, WC, FG and dyslipidaemia. All five biomarkers were, individually, significantly associated with incident diabetes. The ORs for hypoadiponectinaemia, TNF-α R2, A-FABP, IL-6 and hsCRP were 2.62 (P = 0.001), 2.19 (P = 0.004), 2.26 (P = 0.005), 2.25 (P = 0.009) and 2.26 (P = 0.010) respectively.

**Figure 1:**
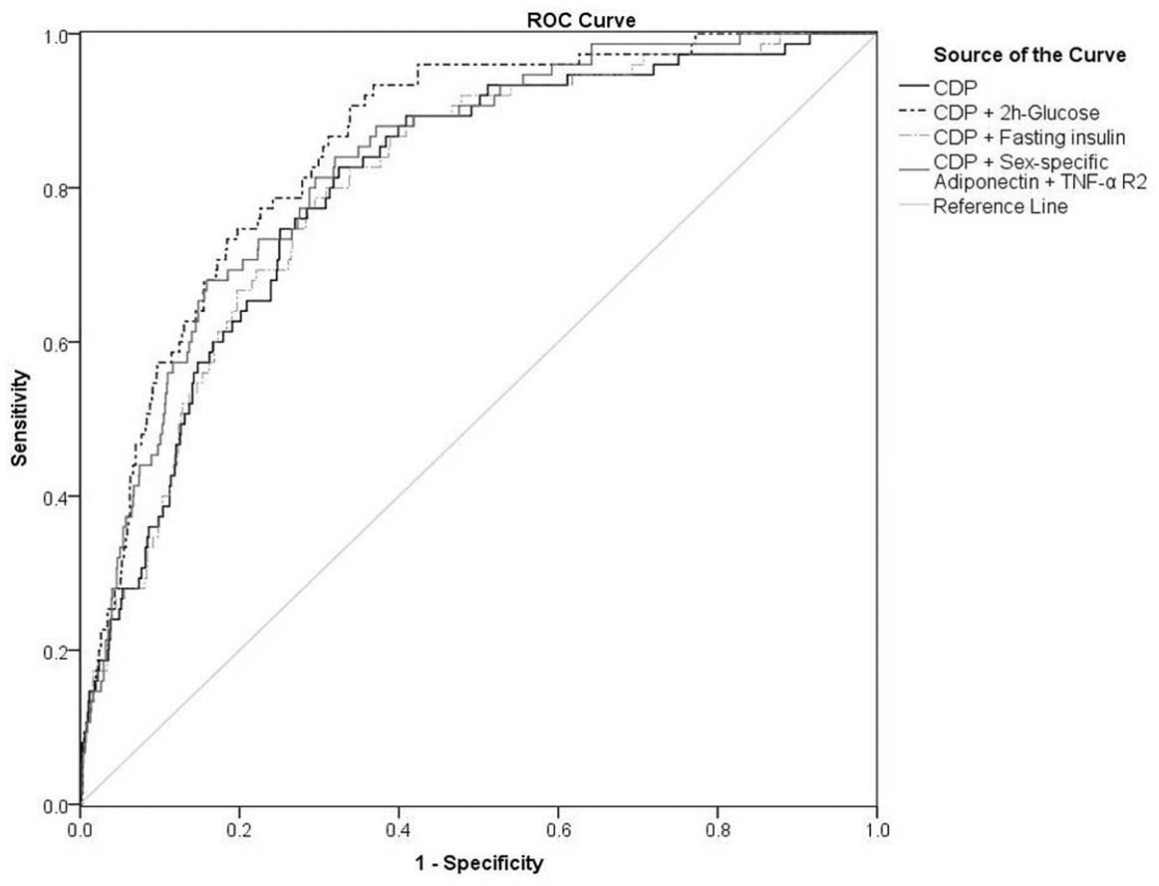
ROC curves for different diabetes prediction models. CDP, clinical diabetes prediction model.

We constructed a clinical diabetes prediction model (CDP) using patients’ baseline demographic parameters and conventional risk factors (age, sex, WC, FG, smoking status, physical activities, family history of diabetes, presence of hypertension and dyslipidaemia). Effect of the addition of biomarkers to the CDP model was shown in Table 2. The largest increment in the log-likelihood of the model was observed after the addition of adiponectin. Addition of each of the other biomarkers to this “CDP + adiponectin” model resulted in further significant increase in log-likelihood (Table 2). Performance of the prediction models was examined by ROC curves analysis. Table 3 showed the comparison of the AUCs of different “CDP + biomarker(s)” models with the baseline CDP model and also the “CDP + 2 h-glucose” model. Adiponectin together with TNF-α R2, when added to the CDP, generated a model with an AUC of 0.830 (95% CI: 0.808–0.850), which was significantly better than the baseline CDP (AUC = 0.802, P = 0.0306) and was comparable to the “CDP + 2 h-glucose” model (AUC = 0.852 [95% CI: 0.831–0.870], P = 0.2954) (Fig. 1) (Table 3). This combination required the smallest number of biomarkers to achieve a performance comparable to 2 h-glucose. Adding fasting insulin level, a marker of insulin resistance, to the CDP did not show significant AUC improvement (AUC = 0.803 [95% CI: 0.780–0.824], P = 0.65). A biomarker risk score, counted by the number of biomarkers predictive of diabetes, i.e. low adiponectin and/or high TNF-α R2, was analyzed by multiple logistic regression as shown in Table 4. The ORs were 2.802 (P = 0.006) and 5.862 (P<0.001) for a score of 1 and 2 respectively. The AUC of the “CDP + biomarker risk score” model was 0.829 (95% CI: 0.808–0.849) which was similar to the “CDP + adiponectin + TNF-α R2” model. The sensitivity and specificity for diabetes prediction of the “CDP + biomarker risk score” model were 70.7% and 83.3% respectively. This model increased the positive predictive value from 21.5% to 31.3% without jeopardizing the negative predictive value (96.4% vs. 97.3%) when compared with the baseline CDP model (Table 5).

**Table 2:** Log-likelihood ratio tests comparing the change before and after addition of biomarkers.

Level	Model	Referent model	-2LL	Change in -2LL	p-value
0	CDP	–	486.254	–	–
1	CDP + **Adiponectin**	CDP	**474.104**	**12.15**	**0.0005**
	CDP + TNF-α R2		477.709	8.545	0.0035
	CDP + A-FABP		478.265	7.989	0.0047
	CDP + IL-6		478.988	7.266	0.0070
	CDP + hsCRP		478.938	7.316	0.0068
2	CDP + Adiponectin + **A-FABP**	CDP + Adiponectin	**465.421**	**8.683**	**0.0032**
	CDP + Adiponectin + **TNF-α R2**		**466.023**	**8.081**	**0.0045**
	CDP + Adiponectin + **IL-6**		**467.771**	**6.333**	**0.0119**
	CDP + Adiponectin + **hsCRP**		**468.742**	**5.362**	**0.0206**

-2LL, -2log-likelihood; p-value (χ^2^, df = 1);

All biomarker levels were sex specific (except for hsCRP).

CDP: Sex, Age, Waist circumference, fasting glucose, hypertension, dyslipidaemia, family history of diabetes, physical activity and smoking status.

TNF-α R2: tumor neurosis factor-alpha receptor 2; hsCRP, high sensitivity C-reactive protein; IL-6, Interleukin-6; A-FABP, adipocyte-fatty acid-binding protein.

**Table 3:** Comparisons of AUCs of different diabetes prediction models.

Level	Model	AUC (95% CI)	P-value *(Referent: CDP)*	P-value *(Referent: 2* *h-glucose)*
0	CDP	0.802 (0.779–0.823)	Referent	–
1	CDP + 2 h-glucose	0.852 (0.831–0.870)	**0.0078**	Referent
	CDP + Fasting insulin	0.803 (0.780–0.824)	0.6512	0.0094
	CDP + Adiponectin	0.816 (0.794–0.837)	0.1375	0.0621
	CDP + TNF-α R2	0.814 (0.792–0.835)	0.1982	0.0723
	CDP + A-FABP	0.809 (0.787–0.830)	0.4304	0.0308
	CDP + hsCRP	0.812 (0.790–0.833)	0.2778	0.0347
	CDP + IL-6	0.807 (0.785–0.829)	0.4992	0.0231
2	**CDP + Adiponectin + TNF-α R2**	0.830 (0.808–0.850)	**0.0306**	**0.2954**
	CDP + Adiponectin + A-FABP	0.825 (0.804–0.846)	0.0633	**0.1820**
	CDP + Adiponectin + IL-6	0.824 (0.802–0.844)	0.0691	**0.1606**
	CDP + Adiponectin + hsCRP	0.824 (0.802–0.844)	0.0938	**0.1618**

AUC, Area under the curve; All biomarker levels were sex-specific except for hsCRP;

CDP: Sex, Age, Waist circumference, fasting glucose, hypertension, dyslipidaemia, family history of diabetes, physical activity and smoking status.

TNF-α R2: tumor neurosis factor-alpha receptor 2; hsCRP, high sensitivity C-reactive protein; IL-6, Interleukin-6; A-FABP, adipocyte-fatty acid-binding protein.

**Table 4:** Multivariate prediction of diabetes according to CDP and biomarker risk score.

	OR (95%CI)	p-value
Men	**0.507 (0.272–0.947)**	**0.033**
Age	**1.042 (1.016–1.068)**	**0.001**
WC	**1.044 (1.013–1.076)**	**0.005**
FG	**3.389 (2.025–5.672)**	**<0.001**
HT	0.757 (0.421–1.360)	0.352
Dyslipidaemia	1.198 (0.657–2.185)	0.556
Family history of DM	1.661 (0.885–3.118)	0.114
Physical activity	0.637 (0.356–1.141)	0.129
Smoking status	1.226 (0.646–2.332)	0.534
Biomarker risk score		**<0.001**
0 = high adiponectin and low TNF-α R2	**Referent**	**–**
1 = either low adiponectin or high TNF-α R2	**2.802 (1.337–5.873)**	**0.006**
2 = low adiponectin and high TNF-α R2	**5.862 (2.601–13.212)**	**<0.001**

WC, waist circumference; FG, fasting glucose; HT, hypertension; TNF-α R2: tumor neurosis factor-alpha receptor 2.

**Table 5:** Performance of different models on diabetes risk prediction.

Model	Accuracy (%)	Sensitivity (%)	Specificity (%)	Positive predictivevalue (%)	Negative predictivevalue (%)
CDP	68.4	82.7	67.5	21.5	97.3
CDP + 2 h-Glucose	67.6	90.7	66.2	22.4	98.5
**CDP + Biomarker risk score**	**82.6**	**70.7**	**83.3**	**31.3**	**96.4**

CDP: sex, age, waist circumference, fasting glucose, hypertension, dyslipidaemia, family history of diabetes, physical activity and smoking status.

Biomarker risk score: (as described in table 4).

Prevalence rate 9.7% [1].

## Discussion

Various serum biomarkers representing the process of adipokine dysregulation and adipose tissue inflammation have been shown to be associated with the development of obesity-related type 2 diabetes. To look for the best predictive obesity-related biomarkers for type 2 diabetes in this 5-year prospective study among Southern Chinese, we focused on biomarkers which were previously shown to be associated with incident diabetes or worsening of glycaemic status in Chinese subjects. Adiponectin [14], A-FABP [13] and hsCRP [8] were therefore selected. IL-6 and TNF-α R2 were also included, being adipokines upstream of CRP in the inflammatory cascades. Individually, low adiponectin, high A-FABP, TNF-α R2 (a surrogate marker of TNF-α), IL-6 or hsCRP at baseline was independently predictive of 5-year diabetes risk in this cohort, after adjustment for nine conventional risk factors, including age, sex, family history of diabetes, smoking, physical inactivity, hypertension, waist circumference, fasting glucose and dyslipidaemia. Each of these biomarkers could increase the likelihood of diabetes development when added to a clinical prediction model comprising these conventional risk factors, with the increase being greatest following the addition of adiponectin. Based on ROC analysis, the combined use of serum adiponectin and TNF-α R2 resulted in a significant enhancement of diabetes prediction by this clinical prediction model, with the enhancement being comparable to that provided by 2-hour plasma glucose, as assessed with a 75 g OGTT.

The association of IL-6 with insulin resistance was suggested by the observation in animal studies that passive immunoneutralisation of IL-6 led to an improved insulin sensitivity in insulin resistant mice with transgenic NFκB activation [22]. Accordingly, elevated levels of IL-6 also predicted an increased risk of diabetes in postmenopausal women [9] and in the current study. On the contrary, however, blocking the action of IL-6 in rheumatoid arthritis patients led to enhanced plasma glucose levels [23], and IL-6 infusion acutely increased insulin-stimulated glucose disposal in humans, probably via AMP-activated protein kinase in skeletal muscles [24]. The role of IL-6 in insulin resistance in humans thus remains to be resolved. It has also been suggested that, as TNF-α can trigger IL-6 release, increased systemic IL-6 levels may reflect enhanced adipose tissue production of TNF-α, the actual driver behind obesity-related insulin resistance [23]. High hsCRP was also shown to be associated with an increased diabetes risk, when analyzed individually, in some previous studies. [8], [25], [26], [27] However, heterogeneity existed as was documented by a recent meta-analysis of 16 published studies [27], with the authors concluding that CRP may not be an independent risk factor for type 2 diabetes, as it is a downstream marker of the inflammatory process, stimulated by pro-inflammatory cytokines including IL-6 and TNF-α, the secretion of which are reciprocally regulated by adiponectin [28]. In the current study, hsCRP and IL-6 were not as useful as TNF-α R2, when used in addition to adiponectin for the prediction of diabetes risk. Similarly, serum A-FABP levels, shown to be associated with various cardiometabolic risk factors in Chinese [13] and Caucasians [29], and independently predictive of diabetes in a CRISPS subcohort [13], appeared to be less useful than TNF-α R2, when used in conjunction with adiponectin, in predicting diabetes development in this larger cohort. The mechanisms underlying the dysglycaemic effect of A-FABP are not fully understood although animal studies suggest several potential mechanisms [28]. In mice, genetic deficiency of A-FABP is associated with enhanced insulin signaling, in part via a reduction of TNF-α, IL-6 and other pro-inflammatory cytokines [28].

TNF-α R2 and adiponectin emerged as the better combination of biomarkers in our diabetes prediction models. TNF-α has been considered as a key mediator of obesity-related insulin resistance because of its increased expression in obesity and its inhibitory effect on insulin receptor signaling [30]. While earlier reports failed to demonstrate the association of serum TNF-α level with diabetes development [10], [12], a more recent study demonstrated that raised serum TNF-α R2 levels, measured as a surrogate marker for TNF-α because of its superior sensitivity and reliability when assayed in frozen plasma, were associated with a modest increase in diabetes risk [9]. Adiponectin, on the other hand, is an adipokine with insulin-sensitizing, anti-inflammatory and vasoprotective properties which has been extensively studied and reviewed in the past decade [28], [31]. Since the first report of its role in diabetes development in a case-control study of Pima Indians [32], an association between low adiponectin and the risk of diabetes has been consistently reported across diverse populations [11], [12], [26], [33], [34], [35]. Adiponectin has also been selected for construction of diabetes prediction models in previous studies [26], [36]. In a German study [26], adiponectin modestly increased the ROC AUC by 0.011 to 0.831 when added to a basic model which included already the inflammatory markers CRP, IL-6, soluble ICAM-1 and soluble E-selectin levels on top of demographic and lifestyle factors, family history of diabetes, blood pressure, lipid levels. In the Inter99 cohort, adiponectin, together with CRP, ferritin, IL-2 receptor A, fasting glucose and insulin, performed similarly as 2-hour plasma glucose (OGTT) or 2-hour insulin in ROC AUC analysis. In this study we demonstrated that only two obesity-related biomarkers, namely adiponectin and TNF-α R2, were required to achieve an effect comparable to 2-hour glucose, when added to a predictive model consisting of non-invasively assessable clinical parameters together with fasting glucose and lipid levels.

Different diabetes prediction models have been published previously. They could be derived from clinical parameters [37], serum biomarkers [36], or a combination of both [38]. The 2-hour post-OGTT plasma glucose has been shown to be the strongest single predictor for diabetes development in Southern Chinese [39]. Although the 2-hour glucose, when used alone, was shown to be inferior to a model derived from readily available clinical variables in the San Antonio Study (77.5% vs. 84.3%) [40], or equivalent to a diabetes prediction score involving six biomarkers [36] in the Inter99 cohort, it is noteworthy that the 2-hour glucose did further improve diabetes prediction based on clinical variables in the San Antonio Study [40], and even more so in the current study. Nonetheless, the OGTT is notorious for being inconvenient, cumbersome and associated with large intra-individual variation in glucose responses. In our pursuit of a potential alternative to 2-hour glucose we aimed at identifying biomarkers comparable to 2-hour glucose in improving the performance of our clinical diabetes prediction model. Fasting insulin, a marker of insulin resistance, was tested but did not result in significant improvement in diabetes risk prediction. In the process of model development, we categorized the biomarkers to low and high levels, with reference to mathematically derived optimal cut-offs, and formulated a simple biomarker risk score by counting the number of biomarkers predictive of diabetes development. As only two biomarkers were needed in our model, the biomarker risk score was simply 0, 1 or 2. This biomarker risk score was also shown to be an independent predictor of diabetes risk in our cohort and improved the performance of the clinical prediction model to a similar level as the addition of 2-hour glucose.

As our study is limited by the relatively small number of subjects with incident diabetes, we have not performed model validation in the current report. In addition, laboratory measurements for adiponectin and TNF-α R2 are still not readily available in clinical practice. However, our postulated diabetes prediction model involved only simple anthropometric measurements and a single fasting blood sample for identifying patients at increased risk of developing type 2 diabetes, which would be more convenient than the conventional oral glucose tolerance test. In summary, our data highlight the potential combined use of serum adiponectin and TNF-α R2 for diabetes prediction model construction in the Chinese population, as an attractive alternative to existing methods which warrant further validation in other populations. Identifying individuals at risk using simple and convenient prediction tools, followed by the commencement of strategic preventive measures, should be a useful approach to halt the epidemic of type 2 diabetes.
